# Resection of Upper-Half of Humerus

**Published:** 1864-04

**Authors:** John Stainback Wilson

**Affiliations:** Surg. Jackson Hospital.


ART. IV.—*Resection of Upper-half of Humerus.* Reported by JOHN STAINBACK WILSON, Surg. Jackson Hospital.

The following case, recently admitted into one of the wards under my charge, is deemed sufficiently interesting to report:

Isaac Sykes, aged 26, a musician in Stuart's Cavalry, was wounded at Gettysburg, 3d July, 1863, and taken prisoner. On 8th of March, 1864, he came under my observation, having arrived on the late flag of truce boat. Wound, gun-shot, the ball passing antero-posteriorly through the upper part of the humerus, just below the axillary folds, and of course fracturing the bone extensively, as the operation will show. Resection was performed on 4th of July, the day after the injury. The whole of the upper-half of the humerus, including the head, was removed.

Present condition: When the muscles are relaxed, the arm dangles by the side, and can be moved about in any and every direction. Indeed, it has very much the appearance of a pasteboard toy or "jumping Jacob," and my first impression was that the limb was a useless appendage, and that amputation would have rendered the condition of the patient more desirable. But, on a closer examination, I found that the functions of the biceps, triceps, and, in fact, of all the principal muscles of the arm, were unimpaired, while all the movements of the fore-arm were *perfect*. When the muscles mentioned were contracted, so as to give a *point d'appui* for the action of the muscles of the fore-arm, all its motions were made with the greatest facility, while those of the arm itself were preserved to a truly wonderful extent. The arm could be carried forward, backward, and adducted with ease.

The removal of the attachment of the deltoid of course interfered with the elevation of the member; but the retention, to a great degree, of all the other movements, and the entire integrity of the functions of the fore-arm, gave the young man a limb which justified him in saying that he "would not take anything for it." He stated that "the Yankee doctors" at first condemned the operation, and "wanted still to cut off his arm." But, fortunately for him, he would not consent.

This triumph of conservative surgery is due to the skill of Surgeon Metcalf, of General Hampton's staff.

The case suggests for consideration the following question: If the whole of the *upper*-half of the humerus can be removed with such good results—if removal of the *lower* part of this bone be the most satisfactory of all the resections—and if resection of the *middle* portion be attended with less difficulty in its performance, and less interference with muscular attachments than in either the upper or lower part, *should not resection be preferred to amputation in almost every case of gun-shot wound of the arm not greatly lacerating the muscles, and involving the principal artery and nerves?*

